# Two-Dimensional Octuple-Atomic-Layer M_2_Si_2_N_4_ (M = Al, Ga and In) with Long Carrier Lifetime

**DOI:** 10.3390/mi14020405

**Published:** 2023-02-08

**Authors:** Yimin Ding, Kui Xue, Jing Zhang, Luo Yan, Qiaoqiao Li, Yisen Yao, Liujiang Zhou

**Affiliations:** 1 Yangtze Delta Region Institute (Huzhou), University of Electronic Science and Technology of China, Huzhou 313001, China; 2 School of Physics, University of Electronic Science and Technology of China, Chengdu 610054, China; 3 Institute of Fundamental and Frontier Science, University of Electronic Science and Technology of China, Chengdu 611731, China

**Keywords:** 2D III-nitride materials, electronic property, optical property, carrier lifetime, first-principles calculations

## Abstract

Bulk III-nitride materials MN (M = Al, Ga and In) and their alloys have been widely used in high-power electronic and optoelectronic devices, but stable two-dimensional (2D) III-nitride materials, except h-BN, have not been realized yet. A new kind of 2D III-nitride material M${}_{2}$Si${}_{2}$N${}_{4}$ (M = Al, Ga and In) is predicted by choosing Si as the appropriate passivation element. The stability, electronic and optical properties of 2D M${}_{2}$Si${}_{2}$N${}_{4}$ materials are studied systematically based on first-principles calculations. The results show that Al${}_{2}$Si${}_{2}$N${}_{4}$ and Ga${}_{2}$Si${}_{2}$N${}_{4}$ are found to be indirect bandgap semiconductors, while In${}_{2}$Si${}_{2}$N${}_{4}$ is a direct bandgap semiconductor. Moreover, Al${}_{2}$Si${}_{2}$N${}_{4}$ and In${}_{2}$Si${}_{2}$N${}_{4}$ have good absorption ability in the visible light region, while Ga${}_{2}$Si${}_{2}$N${}_{4}$ is an ultraviolet-light-absorbing material. Furthermore, the carrier lifetimes of Ga${}_{2}$Si${}_{2}$N${}_{4}$ and In${}_{2}$Si${}_{2}$N${}_{4}$ are as large as 157.89 and 103.99 ns, respectively. All these desirable properties of M${}_{2}$Si${}_{2}$N${}_{4}$ materials make them attractive for applications in electronics and photoelectronics.

## 1. Introduction

As third-generation semiconductors, bulk III-nitride materials, except h-BN, such as InN, GaN, AlN and their alloys, have attracted considerable attention for the application of high-power electronic and optoelectronic devices because of their tunable direct bandgap, high electron saturation mobility, high breakdown voltage and other unique properties [1,2,3,4,5]. Most of these applications are developed based on three-dimensional III-nitride materials, while a small part of them are developed based on zero-dimensional quantum dots or one-dimensional nanowires. Inspired by the fabrication of graphene and its remarkable properties driven by strong electron confinement in a two-dimensional (2D) limit, it is of great significance to study 2D III-nitride materials, which are pivotal to the development of low-power consumption devices and flexible electronic devices.

To date, various research works have been conducted to explore the structural and electronic properties of 2D III-nitride materials. On the one hand, theoretical simulations and calculations show that 2D AlN/GaN/InN monolayers exhibit a graphene-like structure, with each metal atom connected with three N atoms. Their bandgaps are much larger than those of bulk counterparts, and their exciton binding energies are as large as 0.6–1.9 eV [6] due to the sp${}^{2}$ or sp${}^{2}$+sp${}^{1}$ hybridizations. Apart from the graphene-like hexagonal structure, other possible structures for 2D III-nitride monolayer have also been predicted, such as haeckelite GaN (T-GaN) [7], polyporous H-GaN [8] and planar allotropes of AlN [9]. More potential applications have been proposed for the 2D III-nitride monolayers [10,11]. On the other hand, there have been many attempts to experimentally synthesize 2D AlN, GaN or InN monolayers. Unfortunately, the synthesis of stable III-nitride monolayers with graphene-like hexagonal structures has not been realized until now. Although ultrathin 2D AlN, GaN or InN nanosheets have been verified in some limited reports [12,13], the poor stability and strict experimental conditions need to be improved. By contrast, there are some studies reporting the epitaxial growth of 2D III-nitride multilayers sandwiched by graphene and substrate. Robinson and co-workers reported the synthesis of 2D GaN via a migration-enhanced encapsulated growth (MEEG) technique utilizing epitaxial graphene [14], in which the buckled GaN layers were sandwiched by graphene multilayers and SiC substrate. Thereafter, a similar method was used to synthesize 2D AlN or InN layers sandwiched by graphene and SiC substrate [15,16]. Obviously, graphene plays a pivotal role in stabilizing these 2D buckled structures. Decomposition and oxidation might be expected when removing the graphene covering layer. The reasons for the difficulty in preparating 2D III-nitride materials can be attributed to unsaturated dangling bonds on the surfaces of group III-nitrides, large lattice mismatch in heteroepitaxy and the low rate of lateral migration [11].

Recently, Ren and co-workers introduced elemental silicon during chemical vapor deposition growth of nonlayered molybdenum nitride, which enabled the growth of a new 2D material, MoSi${}_{2}$N${}_{4}$ [17]. This is a new general strategy to achieve 2D materials using appropriate elements to passivate the high-energy surfaces of nonlayered materials during growth. Afterwards, a new 2D family MA${}_{2}$Z${}_{4}$ (M = transition mental; A = C, Si and Ge; and Z = N, P and As) was explored, which shows potential applications in photocatalytic catalysts, spintronics and optoelectronics [18,19]. According to this method, new 2D III-nitrides would be designed by choosing an appropriate passivation element, which has not been studied yet.

Herein, 2D III-nitrides M${}_{2}$Si${}_{2}$N${}_{4}$ (M = Al, Ga and In) with octuple atomic layers are predicted based on the first-principles calculations. The mechanical, thermal and dynamic stabilities of M${}_{2}$Si${}_{2}$N${}_{4}$ monolayers are confirmed carefully. Among them, Al${}_{2}$Si${}_{2}$N${}_{4}$ is an indirect bandgap semiconductor with a bandgap of 1.21 eV, while In${}_{2}$Si${}_{2}$N${}_{4}$ is a direct bandgap semiconductor with a bandgap of 1.49 eV. The transition from indirect bandgap in Al${}_{2}$Si${}_{2}$N${}_{4}$ to direct bandgap in In${}_{2}$Si${}_{2}$N${}_{4}$ can be attributed to pd coupling due to the existence of occupied In:d bands. Moreover, 2D M${}_{2}$Si${}_{2}$N${}_{4}$ monolayers show high lightharvesting ability in visible and ultraviolet wavelength regions. Most importantly, the carrier lifetimes of Ga${}_{2}$Si${}_{2}$N${}_{4}$ and In${}_{2}$Si${}_{2}$N${}_{4}$ are calculated to be as large as 157.89 and 103.99 ns, respectively. Our results demonstrate potential applications of M${}_{2}$Si${}_{2}$N${}_{4}$ monolayers in the next-generation optoelectronic technologies.

## 2. Computational Methods

The ground-state electronic properties were calculated using the density functional theory (DFT)-based [20] first-principles method implemented in the Vienna ab initio simulation package (VASP) [21,22]. The generalized gradient approximation (GGA) of Perdew-Burke-Ernzerhof (PBE) functionals [23,24] was adopted to model the exchange–correlation interactions. The electronic interactions were described by projector-augmented-wave (PAW) potentials [22,25]. The cutoff energy for the plane-wave basis was set to be 550 eV, and the Γ-centered 15 × 15 × 1 k-point mesh was adopted to sample the Brillouin zone (BZ) [26,27]. To make the interactions between adjacent layers negligible, the vacuum layer thickness along the *z* direction was built to be ≈ 15 Å. Moreover, the convergence criteria for total energy and atomic forces were 10${}^{-6}$ eV and 0.001 eV/Å, respectively. The phonon spectra were obtained in the framework of density functional perturbation theory (DFPT) by PHONOPY code [28]. The ab initio molecular dynamics (AIMD) simulations using Nosé-Hoover thermostat under the NVT ensemble were used to evaluate the thermal stability within a 4 × 4 × 1 supercell. Some data postprocessing for VASP calculations was performed by VASPKIT code [29]. The carrier lifetimes were calculated using nonadiabatic molecular dynamics (NAMD) simulations with the Hefei-NAMD code [30].

To consider the exciton effects, the PBE energies were corrected by the G${}_{0}$W${}_{0}$ approximation according to Green’s function equations, as implemented in Yambo software [31,32]. The ground-state Kohn-Sham energies and wave functions were obtained with the QUANTUM ESPRESSO (QE) package [33,34]. The norm-conserving Vanderbilt pseudopotentials [35] with a kinetic energy cutoff of 100 Ry and the charge density cutoff of 400 Ry were selected to describe the electron-ion interactions. In order to converge the quasi-particle energy gap, the total number of bands was set to be 12 times the number of valence bands, and the cutoff energy was set to be 20 Ry for the response function in the G${}_{0}$W${}_{0}$ step. The absorption spectra were obtained by the solution of the Bethe-Salpeter equation (BSE) [36], for which the seven highest valence bands and seven lowest conduction bands were set to describe the excitons.

## 3. Results and Discussion

**Figure 1:**
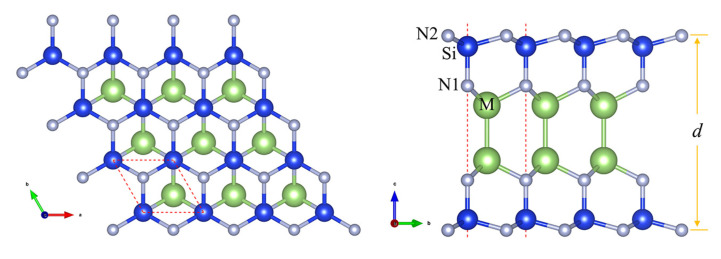
Top (**left** panel) and side (**right** panel) views of M${}_{2}$Si${}_{2}$N${}_{4}$ (M = Al, Ga and In).

**Figure 2:**
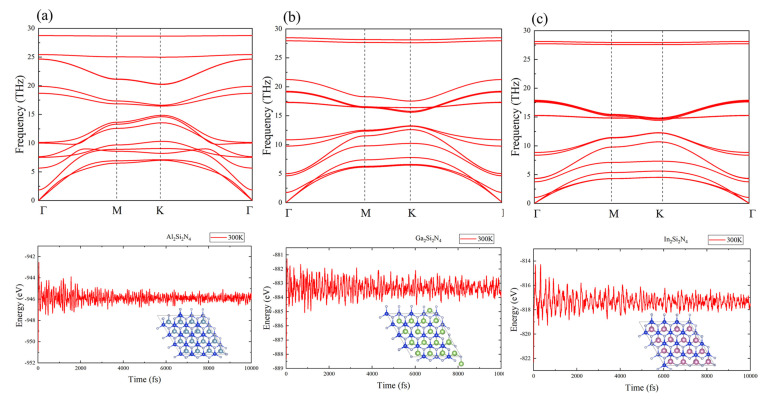
The phonon spectra and energy variations under the AIMD simulations at 300 K of (**a**) Al${}_{2}$Si${}_{2}$N${}_{4}$, (**b**) Ga${}_{2}$Si${}_{2}$N${}_{4}$ and (**c**) In${}_{2}$Si${}_{2}$N${}_{4}$.

The M${}_{2}$Si${}_{2}$N${}_{4}$ (M = Al, Ga and In) monolayers possess a space group of P$\bar{6}$m2 with a D${}_{3h}$ point group symmetry, as shown in Figure 1. There are octuple atomic layers with the sequence of N-Si-N-M-M-N-Si-N in M${}_{2}$Si${}_{2}$N${}_{4}$ monolayer, which can be constructed by intercalating an InSe-type monolayer M${}_{2}$N${}_{2}$ into the middle of two Si-N bilayers. There are two kinds of N atoms, named N1 and N2, based on the difference of coordination number. Furthermore, the stability of M${}_{2}$Si${}_{2}$N${}_{4}$ monolayers are conformed by the calculations of phonon spectra, elastic stiffness constants and AIMD sumulations. Firstly, two independent elastic constants for 2D hexagonal crystals, *C*${}_{11}$ and *C*${}_{12}$, are calculated based on the strain energy method [37], as shown in Table 1. The calculated *C*${}_{11}$ and *C*${}_{12}$ obviously meet the elastic stability criteria, i.e., *C*${}_{11}$ > 0 and *C*${}_{11}$ > |*C*${}_{12}$|. The *C*${}_{11}$ of M${}_{2}$Si${}_{2}$N${}_{4}$ monolayer is as large as 459–572 N/m, even larger than that of graphene (340 N/m) [38]. Secondly, the phonon spectra of M${}_{2}$Si${}_{2}$N${}_{4}$ verify their dynamic stability because of the absence of imaginary frequency modes, as shown in Figure 2. Lastly, no signs of structural fracture and small energy fluctuations during AIMD simulations at the temperature range from 300 to 1000 K demonstrate the thermal stability of M${}_{2}$Si${}_{2}$N${}_{4}$ monolayers (Figure 2 and Figure S1 in Supplementary Materials). In brief, we predict new kinds of 2D material M${}_{2}$Si${}_{2}$N${}_{4}$ and confirm their stabilities. Next, their electronic and optical properties are studied in detail.

**Figure 3:**
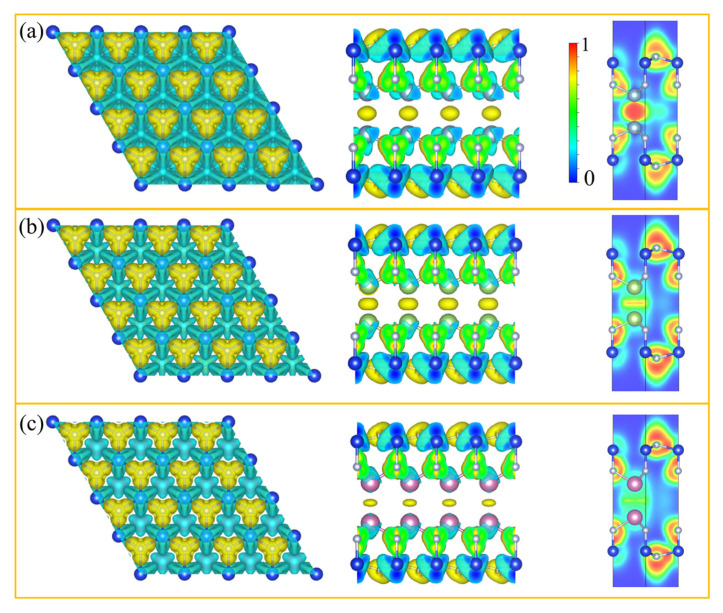
The top and side views of the deformation electron density and electron localization function (ELF) map sliced perpendicularly to (110) direction for (**a**) Al${}_{2}$Si${}_{2}$N${}_{4}$, (**b**) Ga${}_{2}$Si${}_{2}$N${}_{4}$ and (**c**) In${}_{2}$Si${}_{2}$N${}_{4}$. The isovalue is set to be 0.01 e/Bohr${}^{3}$. The deformation electron density is defined as the M${}_{2}$Si${}_{2}$N${}_{4}$ monolayer total charge density, subtracting the sum of isolated atoms. The charge accumulation and depletion regions are shown in yellow and blue, respectively.

The relaxed lattice constant of M${}_{2}$Si${}_{2}$N${}_{4}$ (M = Al, Ga and In) is in the range of 2.97–3.14 Å (Table 1). To achieve deep insights into the bonding nature of M${}_{2}$Si${}_{2}$N${}_{4}$, we then calculate the deformation electron density and electron localization function (ELF) (Figure 3). We can find obvious charge accumulation around N atoms and in the middle of metal atoms, while charge depletion mainly occurs around Si and metal atoms due to the large electronegativity difference between N and Si/M atoms. The ELF plots show that N–Si bonds have mainly covalent bonding interactions. It is worth noting that N–M bonds switch from mainly covalent bonding in Al${}_{2}$Si${}_{2}$N${}_{4}$ to a mixture of covalent and ionic bonds in Ga${}_{2}$Si${}_{2}$N${}_{4}$ and In${}_{2}$Si${}_{2}$N${}_{4}$. The Al–Al, Ga–Ga and In–In bond distances are 2.62, 2.47 and 2.78 Å, respectively, which are shorter than those in bulk Al (2.86 Å), Ga (2.53 Å) and In (3.31 Å). Moreover, Bader [39] charges calculations (Table S1) show that the numbers of electrons transferred from the metal atoms to surrounding atoms are 1.65, 1.06 and 0.97 |*e*| in Al${}_{2}$Si${}_{2}$N${}_{4}$, Ga${}_{2}$Si${}_{2}$N${}_{4}$ and In${}_{2}$Si${}_{2}$N${}_{4}$, respectively. At the same time, electrons transferred to N1 atoms decrease from 2.37 |*e*| in Al${}_{2}$Si${}_{2}$N${}_{4}$ to 1.79 (1.71) |*e*| in Ga${}_{2}$Si${}_{2}$N${}_{4}$ (In${}_{2}$Si${}_{2}$N${}_{4}$). For N2 and Si atoms, transferred electrons are almost the same in the three systems.

**Figure 4:**
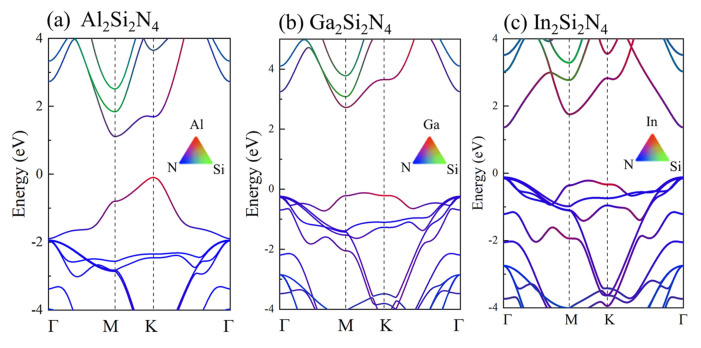
The projected electronic band structure and density of states (DOS) of (**a**) Al${}_{2}$Si${}_{2}$N${}_{4}$, (**b**) Ga${}_{2}$Si${}_{2}$N${}_{4}$ and (**c**) In${}_{2}$Si${}_{2}$N${}_{4}$. The Fermi energy is set to 0 eV.

**Figure 5:**
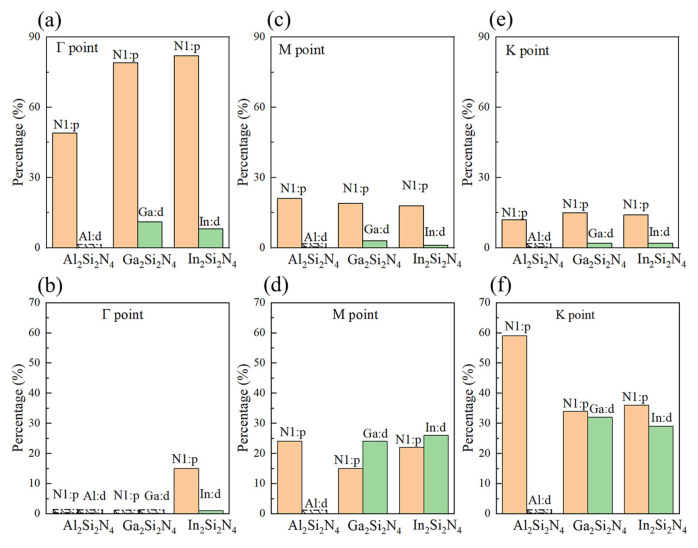
Atomic orbital components of the highest valence band and the lowest conduction band at Γ, (**a**,**b**) M (**c**,**d**) and K points (**e**,**f**). The blank boxes indicate zero.

The projected electronic band structures and density of states are calculated based on DFT-PBE level, as shown in Figure 4. For Al${}_{2}$Si${}_{2}$N${}_{4}$, it is an indirect bandgap semiconductor (1.21 eV) with valence band maximum (VBM) located at the K point and conduction band minimum (CBM) located at the M point. The conduction (CBE) and valence (VBE) band edges are mainly determined by N1:p states and Al:p states, respectively (Figure S2). For Ga${}_{2}$Si${}_{2}$N${}_{4}$, VBM is located at a special point near K, and CBM is located at the M point, resulting an indirect bandgap of 2.84 eV, while the CBE and VBE are mainly determined by Ga:p and Ga:p,d states hybridized with N1:p, respectively. Most importantly, In${}_{2}$Si${}_{2}$N${}_{4}$ is a direct-bandgap (1.49 eV) semiconductor, with both CBM and VBM located at the Γ point, while CBE mainly originates from the hybridized In:s,p and N1:s,p states, and VBE is mainly determined by N1:p states. The band edge switch is clearly related with the occupied d orbitals in Ga${}_{2}$Si${}_{2}$N${}_{4}$ and In${}_{2}$Si${}_{2}$N${}_{4}$, which are absent in the Al${}_{2}$Si${}_{2}$N${}_{4}$ monolayer (Figure S3). In order to explore the origin of the indirect-to-direct bandgap transfer, we plot the atomic orbital components of the highest valence band and the lowest conduction band at Γ, M and K points for three M${}_{2}$Si${}_{2}$N${}_{4}$ monolayers, as shown in Figure 5. From Figure 5a, we can see that the highest valence band at Γ is composed of N1:p states in Al${}_{2}$Si${}_{2}$N${}_{4}$ and hybridized N1:p and M:d states in Ga${}_{2}$Si${}_{2}$N${}_{4}$ (In${}_{2}$Si${}_{2}$N${}_{4}$). The pd couplings only emerging in Ga${}_{2}$Si${}_{2}$N${}_{4}$/In${}_{2}$Si${}_{2}$N${}_{4}$ would push valence energy level at Γ point up [40]. Although the pd couplings would also push the energy level at K and M points up, the energy increase at Γ might be larger than that at the K and M points because of the higher percentage of p and d orbitals in Γ compared with K and M points (Figure 5c,e), finally resulting in the valence energy level at Γ to be the highest among the three points, i.e., leading Γ to be VBM in In${}_{2}$Si${}_{2}$N${}_{4}$. On the contrary, the pd coupling would push the conduction energy levels at M and K points up, leading the conduction energy level at Γ to be the lowest among the three points, i.e., Γ becoming CBM in In${}_{2}$Si${}_{2}$N${}_{4}$. In one word, the existence of occupied d orbitals plays a key role in determining the directness or indirectness of bandgap in M${}_{2}$Si${}_{2}$N${}_{4}$.

**Figure 6:**
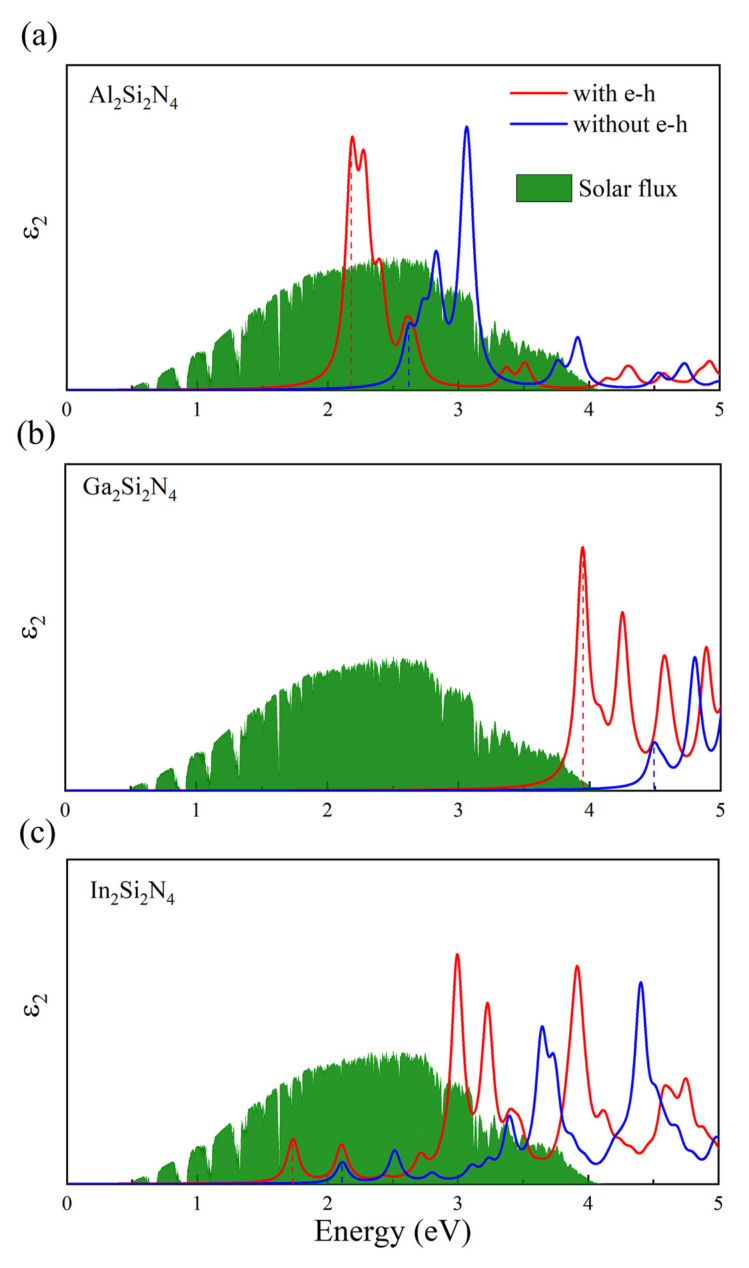
Imaginary part of the dielectric function of (**a**) Al${}_{2}$Si${}_{2}$N${}_{4}$, (**b**) Ga${}_{2}$Si${}_{2}$N${}_{4}$ and (**c**) In${}_{2}$Si${}_{2}$N${}_{4}$. The spectra with e-h interaction and without e-h interaction were calculated on the basis of G${}_{0}$W${}_{0}$-BSE and G${}_{0}$W${}_{0}$-RPA methods, respectively. The standard AM1.5 G solar flux is shown to reflect the light capture ability.

Generally, the exciton binding energy is very large in ultrathin 2D materials, such as 0.63 eV in MoS${}_{2}$ [41], 0.7 eV in InSe [42] and 0.6–2.0 eV in graphene-like *h*-MN (M = B, Al, Ga and N) [6]. Considering the relatively large thickness of M${}_{2}$Si${}_{2}$N${}_{4}$, it is necessary to study the relationship between exciton binding energy and the thickness of 2D materials. Based on GW-BSE methods, we calculate the quasi-particle bandgaps, exciton binding energy (Table 1) and imaginary parts of the dielectric function (Figure 6) of M${}_{2}$Si${}_{2}$N${}_{4}$ monolayers. The first prominent peak defined as the optical gap *E*${}_{\mathrm{opt}}$ is located at 2.18, 3.95 and 1.73 eV for Al${}_{2}$Si${}_{2}$N${}_{4}$, Ga${}_{2}$Si${}_{2}$N${}_{4}$ and In${}_{2}$Si${}_{2}$N${}_{4}$, respectively. This excitonic peak corresponds to the interband transition from the highest valence band to the lowest conduction band at the K, M and Γ points for Al${}_{2}$Si${}_{2}$N${}_{4}$, Ga${}_{2}$Si${}_{2}$N${}_{4}$ and In${}_{2}$Si${}_{2}$N${}_{4}$, respectively. Furthermore, the exciton binding energies are calculated to be 0.44, 0.54 and 0.38 eV. It is clear that the larger thickness of materials (Table 1) results in smaller exciton binding energy. From Figure 6, the absorbance spectra of Al${}_{2}$Si${}_{2}$N${}_{4}$ and In${}_{2}$Si${}_{2}$N${}_{4}$ have a large overlap with the AM1.5G solar flux in the visible wavelength region, demonstrating their potential application in detectors or solar cells. Moreover, the 2D Ga${}_{2}$Si${}_{2}$N${}_{4}$ monolayer shows high lightharvesting ability in the ultraviolet wavelength region.

**Figure 7:**
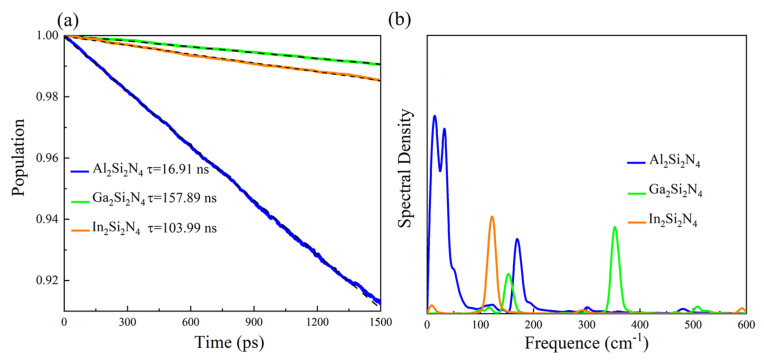
Excited–state carriers’ population decay (**a**) and spectral densities (**b**) of M${}_{2}$Si${}_{2}$N${}_{4}$.

Apart from good lightharvesting ability, the lifetime of excited carriers also plays an important role in the power conversion efficiency in solar cell devices. Thus, we calculate the photogenerated carrier lifetimes driven by nonadiabatic (NA) processes in M${}_{2}$Si${}_{2}$N${}_{4}$ monolayers. According to Fermi’s golden rule and Marcus theory [43,44], a larger band gap, a weaker nonadiabatic coupling (NAC) and a shorter pure-dephasing time would result in a smaller e-h recombination rate and longer carrier lifetime. During the nonequilibrium NA processes, the extra energy of electrons is transferred to the involved nuclei triggered by inelastic electron-phonon couplings, dissipating as heat. The eh recombination rate is proportional to the square of the NAC constant [45], which is determined by the overlap between the initial and final electronic states and the phonon velocity [46]. The calculated NAC is 0.97, 0.55 and 0.30 meV for Al${}_{2}$Si${}_{2}$N${}_{4}$, Ga${}_{2}$Si${}_{2}$N${}_{4}$ and In${}_{2}$Si${}_{2}$N${}_{4}$, respectively. Large NAC would result in high e-h recombination rate and short carrier lifetime. To analyze the phonon modes that drive e-h recombination, the vibrational spectral densities calculated by a Fourier transform (FT) of gap autocorrelation functions (ACFs) are plotted in Figure 7b. For the Al${}_{2}$Si${}_{2}$N${}_{4}$ monolayer, the low vibration frequencies in the range of 10–40 cm${}^{-1}$ participate in the nonradiative e-h recombination, and the largest spectral densities reflect stronger electron-phonon (e-ph) interactions, leading to the largest NAC among the three materials. For Ga${}_{2}$Si${}_{2}$N${}_{4}$ and In${}_{2}$Si${}_{2}$N${}_{4}$, the dominating peak frequencies are located at 125 and 350 cm${}^{-1}$, respectively.

In addition to inelastic electron-phonon couplings, elastic electron-phonon coupling also has an impact on the recombination of photogenerated carriers, which results in a loss of the phase relationship in a quantum-mechanical superposition between VBM and CBM [46]. The pure-dephasing functions are shown in Figure S4, and the dephasing times T of Al${}_{2}$Si${}_{2}$N${}_{4}$, Ga${}_{2}$Si${}_{2}$N${}_{4}$ and In${}_{2}$Si${}_{2}$N${}_{4}$ are 25.4, 13.0 and 9.6 fs, respectively. For Al${}_{2}$Si${}_{2}$N${}_{4}$, the initial un-normalized ACF (uACF) value is the smallest among the three materials (Figure S4), corresponding to the longest pure-dephasing time. However, a shorter pure-dephasing time would lead to a smaller e-h recombination rate and thus longer carrier lifetime. Taking into consideration the above inelastic and elastic factors, we obtain the decay of the carrier populations through the bandgap for three M${}_{2}$Si${}_{2}$N${}_{4}$ monolayers, as shown in Figure 7a. Additionally, the carrier lifetime is evaluated by a short-time linear fitting to the exponential decay, *f*(t) = exp(−t/τ) ≈ 1 − t/τ. At last, the e-h recombination lifetimes are calculated to be 16.91, 157.89 and 103.99 ns for Al${}_{2}$Si${}_{2}$N${}_{4}$, Ga${}_{2}$Si${}_{2}$N${}_{4}$ and In${}_{2}$Si${}_{2}$N${}_{4}$, respectively. Two-dimensional Ga${}_{2}$Si${}_{2}$N${}_{4}$ has the longest carrier lifetime of 157.89 ns, due to the largest bandgap, relatively small NAC and dephasing time (Table S2). Most importantly, the simulated carrier lifetime in Ga${}_{2}$Si${}_{2}$N${}_{4}$ and In${}_{2}$Si${}_{2}$N${}_{4}$ reaches a one hundred nanosecond level, which is much larger than that in previous reported materials, such as MoS${}_{2}$ (0.39 ns) [47], 1T-Ti${}_{2}$OF${}_{2}$ (2.8 ns) [48], MAPbI${}_{3}$ perovskite (12.0 ns) [49], bulk GaN (40 ns) [50] and bulk InN [51]. The long carrier lifetime of 2D M${}_{2}$Si${}_{2}$N${}_{4}$ highlights their potential in optoelectronic and photovoltaic applications.

## 4. Conclusions

In this work, a new kind of 2D material M${}_{2}$Si${}_{2}$N${}_{4}$ (M = Al, Ga and In) with octuple atomic layers is predicted and studied based on first-principles calculations. Firstly, three 2D M${}_{2}$Si${}_{2}$N${}_{4}$ monolayers show high mechanical, thermal and dynamic stabilities. Secondly, Al${}_{2}$Si${}_{2}$N${}_{4}$ and Ga${}_{2}$Si${}_{2}$N${}_{4}$ have an indirect bandgap of 1.21 and 2.84 eV, respectively, while In${}_{2}$Si${}_{2}$N${}_{4}$ is a direct semiconductor with a bandgap of 1.49 eV. The indirect-to-direct bandgap transition in M${}_{2}$Si${}_{2}$N${}_{4}$ is analyzed and attributed to pd couplings due to the existence of occupied Ga:d or In:d orbitals. Thirdly, Al${}_{2}$Si${}_{2}$N${}_{4}$ and In${}_{2}$Si${}_{2}$N${}_{4}$ have strong optical absorption in the visible light region, while Ga${}_{2}$Si${}_{2}$N${}_{4}$ shows high lightharvesting ability in the ultraviolet light region. The exciton binding energy is calculated to be 0.44, 0.54 and 0.38 eV for Al${}_{2}$Si${}_{2}$N${}_{4}$, Ga${}_{2}$Si${}_{2}$N${}_{4}$ and In${}_{2}$Si${}_{2}$N${}_{4}$, respectively, which is a relatively small value in the 2D materials family. Most importantly, the carrier lifetimes of Ga${}_{2}$Si${}_{2}$N${}_{4}$ and In${}_{2}$Si${}_{2}$N${}_{4}$ are as large as 157.89 and 103.99 ns, respectively. Overall, these attractive properties make 2D M${}_{2}$Si${}_{2}$N${}_{4}$ materials promising candidates for optoelectronic and photovoltaic applications.

## Figures and Tables

**Table 1:** The calculated lattice constant *a*, thickness *d*, elastic constants *C*${}_{11}$/*C*${}_{12}$, energy gap *E*${}_{g}$, optical gap *E*${}_{\mathrm{opt}}$ and exciton binding energy *E*${}_{b}$ from GW-BSE methods for M${}_{2}$Si${}_{2}$N${}_{4}$ monolayers.

	*a* (Å)	*d* (Å)	*C*${}_{11}$/*C*${}_{12}$ (N/m)	*E*${}_{g}$ (eV)	*E*${}_{\mathrm{opt}}$ (eV)	*E*${}_{b}$ (eV)
Al${}_{2}$Si${}_{2}$N${}_{4}$	2.97	8.8	571.8/124.5	2.62 (K)	2.18	0.44
Ga${}_{2}$Si${}_{2}$N${}_{4}$	3.01	8.7	541.5/128.3	4.49 (M)	3.95	0.54
In${}_{2}$Si${}_{2}$N${}_{4}$	3.14	9.3	458.7/119.6	2.11 (Γ)	1.73	0.38

## Data Availability

The data presented in this study are available on request from the corresponding author.

## Supplementary Materials

The following supporting information can be downloaded at: https://www.mdpi.com/article/10.3390/mi14020405/s1, Figure S1: Energy variations under the AIMD simulations at 1000 and 1200 K of (a) Al2Si2N4, (b) Ga2Si2N4 and (c) In2Si2N4. Figure S2: The projected density of states (PDOS) of (a) Al2Si2N4, (b) Ga2Si2N4 and. (c) In2Si2N4. Figure S3: The density of states for Ga:d and In:d in Ga2Si2N4 and In2Si2N4. Figure S4: Pure-dephasing functions and unnormalized autocorrelation functions of the electronic energy gap fluctuations for (a) Al2Si2N4, (b) Ga2Si2N4 and (c) In2Si2N4. Table S1: The calculated Bader charge and work function for M2Si2N4. Positive values mean electron loss, and negative values mean electron gains. Table S2: The calculated bandgap Eg, average NAC, pure-dephasing time T and nonradiative electron-hole recombination time τ in M2Si2N4.
