# Supplementary material for: Two-Dimensional Octuple-Atomic-Layer M2Si2N4 (M = Al, Ga and In) with Long Carrier Lifetime
Source: Micromachines (Basel). 2023 Feb 8;14(2):405. doi: 10.3390/mi14020405 (PMC9966885; doi:10.3390/mi14020405)
Supplement: Supplementary file 1 [file micromachines-14-00405-s001.zip › micromachines-2099926-supplementary.pdf]

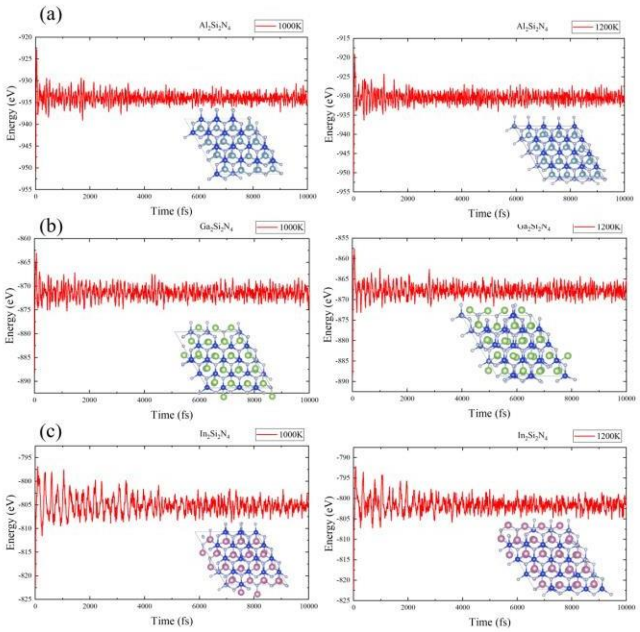

Figure S1. Energy variations under the AIMD simulations at 1000 and 1200 K of (a)$Al_2Si_2N_4$, (b) $Ga_2Si_2N_4$ and (c) $In_2Si_2N_4$.

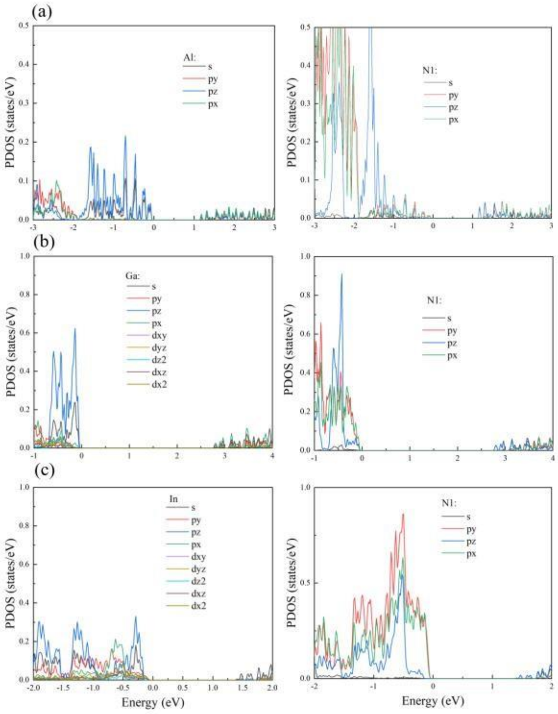

Figure S2. The projected density of states (PDOS) of (a) Al2Si2N4, (b) Ga2Si2N4 and. (c) $In_2Si_2N_4$.

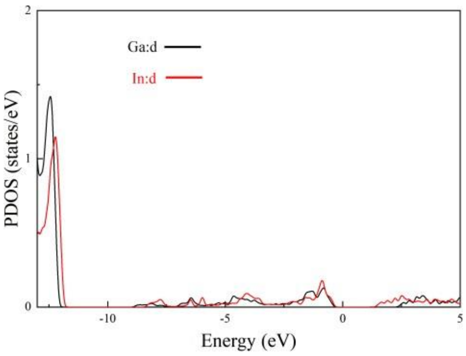

Figure S3. The density of states for Ga:d and In:d in Ga2Si2N4 and In2Si2N4.

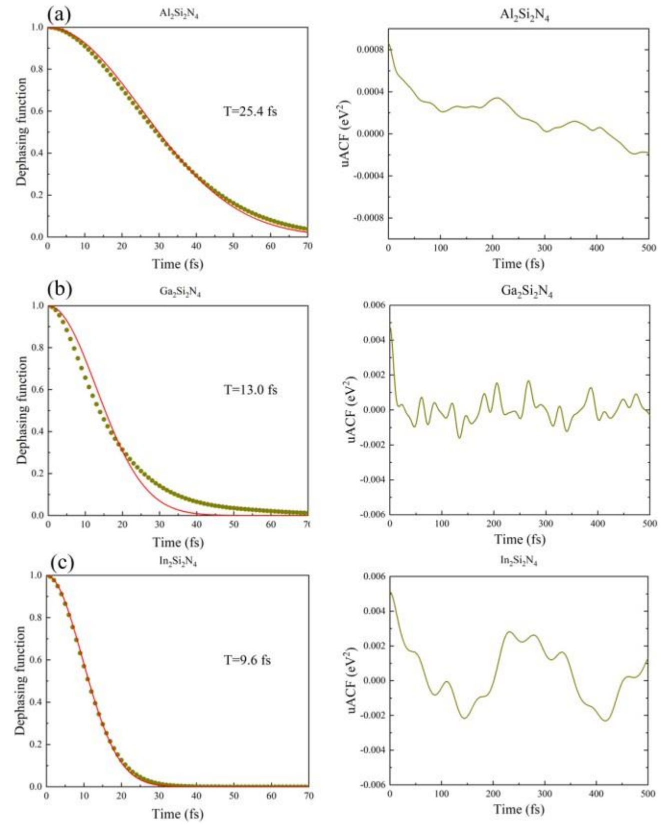

Figure S4. Pure-dephasing functions and unnormalized autocorrelation functions of the electronic energy gap fluctuations for (a) $Al_2Si_2N_4$, (b) $Ga_2Si_2N_4$ and (c) $In_2Si_2N_4$.

Table S1. The calculated Bader charge and work function for $M_2Si_2N_4$. Positive values meanelectron loss, and negative values mean electron gains.

**Charge (e)**	**$Al_2Si_2N_4$**	**$Ga_2Si_2N_4$**	**$In_2Si_2N_4$**
Al/Ga/In	1.65	1.06	0.97
N1	-2.37	-1.79	-1.71
Si	2.94	2.94	2.87
N2	-2.22	-2.20	-2.13
Workfunction(eV)	4.06	6.45	6.43

Table S2. The calculated bandgap E_g, average NAC, pure-dephasing time T and nonradiativeelectron-hole recombination time τ in $M_2Si_2N_4$.

	**$Al_2Si_2N_4$**	**$Ga_2Si_2N_4$**	**$In_2Si_2N_4$**
E_g (eV)	1.21	2.84	1.49
NAC (meV)	0.97	0.55	0.30
T (fs)	25.4	13.0	9.6
τ (ns)	16.91	157.89	103.99
